# Design and numerical simulation of a semi-cast-in-situ synthetic material sports surface on shock absorption performance optimization

**DOI:** 10.1371/journal.pone.0329393

**Published:** 2025-08-01

**Authors:** Hong Wang, Pan Zhang, Weitao Zheng, Gan Liu, Yong Ma, Rui Han

**Affiliations:** 1 College of Intelligent Sports Engineering, Wuhan Sports University, Wuhan, China; 2 Research Center of Sports Equipment Engineering Technology of Hubei Province, Wuhan Sports University, Wuhan, China; 3 Key Laboratory of Sports Engineering of General Administration of Sport of China, Wuhan Sports University, Wuhan, China; 4 Comprehensive Administrative Law Enforcement Team of Street Office, Daguan District, Anqing City, Anhui Province, China; Chongqing University Three Gorges Hospital, CHINA

## Abstract

The persistent issue of moisture-induced “heave” in synthetic sports surfaces can affect athlete safety and surface performance. The objective of the study was to design an innovative synthetic material athletic track structure that mitigates adhesive failure between the synthetic layer and the cement base due to underground moisture. The new structure ensures field safety and meets biomechanical requirements for performance and shock absorption. Numerical simulation methods are employed to analyze the shock absorption performance of the synthetic material track and field facility, incorporating the new structure, and subsequently to propose optimization strategies for the structural design. The optimal structure adopted a circular casting hole design, with a cast-in-situ surface layer thickness of 12 mm, a prefabricated surface layer thickness of 6 mm, a hole diameter of 45 mm, and a hole spacing of 80 mm arranged in a square pattern. The results indicated that the proposed structure not only met the required standards for shock absorption, but also offered a promising solution to the issue of moisture-induced “heave” prevalent in traditional sports surfaces. The study provided important theoretical support and practical guidance for the scientific and efficient construction of athletic tracks.

## Introduction

Synthetic sports surfaces are widely adopted in modern running tracks due to their excellent mechanical properties and ability to support athletic performance [1]. Multiple international standards have been established to ensure their durability, safety and biomechanical requirements for outdoor sports scenarios [2,3]. Despite these advantages, a common failure mode known as “heave” occurs in practical applications. The phenomenon is caused by moisture accumulation at the interface between the synthetic layer and the rigid base, typically cement or asphalt base [4,5]. The moisture becomes trapped when the bond is excessively rigid or poorly ventilated, resulting in swelling, interfacial delamination, and eventual surface cracking under thermal and humidity fluctuations. These defects compromise surface integrity and reduce shock absorption capacity, increasing the risk of sports-related lower limb injuries [6]. Although advances in athletic footwear have partially mitigated these biomechanical risks [7,8], durable and resilient surfaces remain essential for injury prevention and performance enhancement.

Many studies primarily focus on enhancing the impact absorption performance of traditional synthetic sports surfaces through experimental and numerical simulation methods [9,10]. The enhancements are typically achieved by adjusting factors such as surface thickness of synthetic sports surfaces or material composition [11–13]. Some studies proposed structural innovations, such as honeycomb layers or drainage integration [14–17], yet most remain in conceptual or lab-scale stages without full-scale validation. There is a lack of empirical evidence linking surface structure to real-world anti-“heave” performance and shock absorption effectiveness.

The study aims to propose a novel semi-cast-in-situ synthetic track surface structure that combines a prefabricated layer and a cast-in-situ layer. The structure allows for pressure release through unbonded gaps and is designed to enhance shock absorption while mitigating moisture-induced “heave”. This study will combine experimental measurement of material parameters with numerical simulations using the finite element method to analyze the shock absorption performance of the new structure. Based on the simulation results, the surface layer design will be optimized. Finally, the optimized surface structure will be experimentally validated for its feasibility and practical applicability, providing new insights and guidance for the design optimization of synthetic sports surfaces.

## Methods and methods

### Structural design

**Fig 1:**
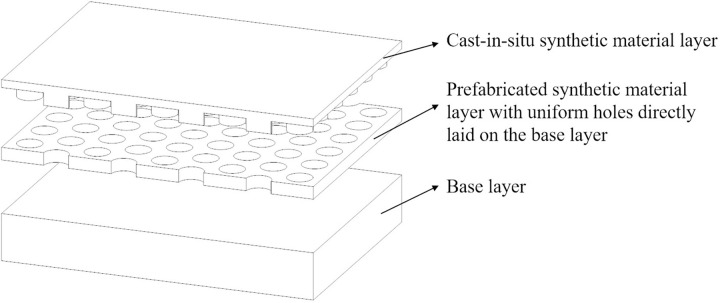
Structural design of semi-cast-in-situ synthetic material sports field.

A semi-cast-in-situ track structure was developed to mitigate moisture-induced “heave” in synthetic sports surfaces. The system consists of prefabricated sheets containing regularly spaced holes, which are directly laid on the rigid concrete base without adhesive bonding at the interface (Fig 1). Each sheet is sized to match the standard width of a running track, allowing for efficient modular installation. After placement, liquid synthetic material is poured into the holes, filling the voids and bonding with the underlying base to form cast-in-situ columns that provide localized mechanical interlocking and vertical fixation. As a result, the structure features a partially bonded configuration: the prefabricated layer remains unbonded to enable lateral moisture diffusion, while the cast-in-situ anchors prevent sliding and “heave”. This hybrid bonding strategy facilitates the release of moisture or vapor pressure through the unbonded interface, helping to prevent the formation of “heave”. Concurrently, the cast-in-situ anchors enhance structural stability and resist displacement under repeated loading.

### Shock absorption

**Fig 2:**
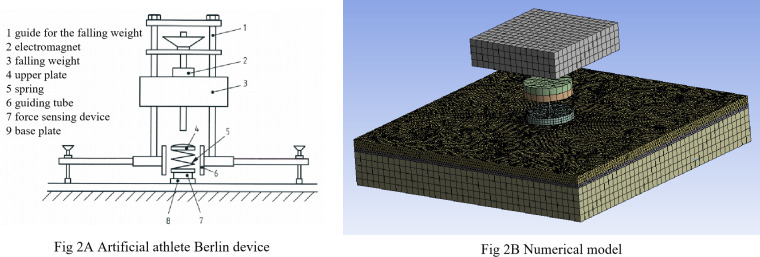
Artificial athlete Berlin.


The surface’s deformability under impact, or compliance, affects how forces are transmitted through the lower limbs. Increased compliance extends the impact time and reduces peak forces, helping to lower injury risk [11,18,19]. Shock absorption refers to the surface’s ability to reduce impact force [20–22]. It is measured by force reduction (FR), defined as the percentage drop in peak force compared to a rigid concrete surface, as shown in Eq (1). Higher FR values indicate better cushioning performance. According to EN 14808, synthetic track surfaces must achieve an FR between 35% and 50% [3], tested using an artificial athlete Berlin device (Fig 2A). Adequate shock absorption will help lower internal stresses during repeated loading, which may reduce surface cracking and indirectly mitigate moisture-induced “heave”.

$$FR=\left(1-\frac{F_{t}}{F_{r}}\right)\times 100$$ (1)

Where: FR is the force reduction, expressed as a percentage(%); $F_{t}$ is the measured maximum peak force for the test piece, N; $F_{r}$ is the measured maximum peak force for the concrete, N.

### Finite element analysis

#### Sports surface and artificial athlete Berlin model.

The simulation model of the synthetic sports surface included an 18 mm semi-cast-in-situ surface over a 50 mm cement concrete base in the study. The surface layer consisted of a 6 mm prefabricated sheet and a 12 mm cast-in-situ layer that filled holes in the prefabricated sheet. The model area was 400 × 400 mm. In the numerical model, some assumptions were made: (1) The cast-in-situ synthetic material was fully bonded within the holes; (2) It was fully bonded to the concrete base at those locations; (3) The remaining interface between the prefabricated layer and the base was modeled as a frictionless, non-bonded contact, replicating the vapor-relief structure; (4) The normal contact pressure was assumed to be uniformly distributed across the impact area to simplify the load condition. In a previous study, bonding conditions between surface layers and rigid substrates were systematically analyzed using finite element methods, providing a foundation for the current model simplification and future refinements [5].

The impact process was simulated in ANSYS (Fig 2B). A 20 kg rigid mass with an initial velocity of 1.04 m/s represented the falling weight. Springs (1.46 kg and 0.518 kg) with stiffness values of 2 kN/mm and 1 MN/mm modeled the spring and the load cell. A 0.651 kg rigid body represented the test foot. The surface material was characterized as hyperelastic, and the base as linear elastic (Young’s modulus of 30 GPa, Poisson’s ratio of 0.15, and a density of 2300 kg/m^3^) [18]. Normal contact was modeled using the penalty method to allow realistic contact interaction during impact.

#### Synthetic material model.

To characterize the mechanical properties of the synthetic materials, quasi-static uniaxial tensile tests were performed using a WDW-1D microcomputer-controlled electronic universal testing machine. The synthetic materials were assumed to exhibit rubber-like behaviour, and testing was conducted based on standard methods for rubber materials (ISO 37: 2005) [19]. Dumbbell-shaped specimens were used [23], and a tensile rate of 100 mm/min was applied. The test was terminated when the sample was pulled apart. To ensure experimental accuracy, five different samples were tested under controlled conditions at a temperature of 23 ± 2°C. The final test data were obtained by averaging the values from all samples.

Considering the expected behaviour of the synthetic materials, the Mooney-Rivlin hyperelastic model was used to describe their constitutive properties [18,19]. For uniaxial deformation, assuming the materials to be isotropic and incompressible, the nominal stress-strain relationship of the 3-Parameter Mooney-Rivlin Model was given in Eq (2).


$$\psi =C_{10}\left({\bar{I}}_{1}-3\right)+C_{01}\left({\bar{I}}_{2}-3\right)+C_{11}\left({\bar{I}}_{1}-3\right)\left({\bar{I}}_{2}-3\right)+\frac{1}{d}{\left(J-1\right)}^{2}$$ (2)

**Fig 3:**
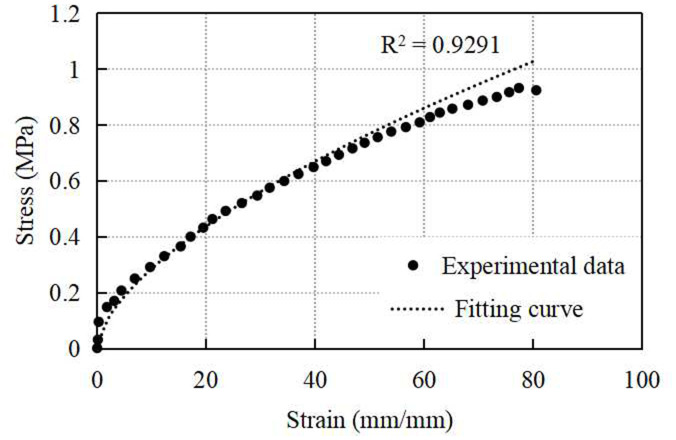
Stress-strain curve of quasi-static uniaxial tensile test.

Where: ψ was the strain energy; ${\bar{I}}_{1},\;{\bar{I}}_{2}$ were the deviatoric first and second principal invariants; $C_{10},\;C_{01},\;C_{11}$ were material constants which were confirmed by the experimental data (Fig 3); d was material incompressibility parameter; J was the Jacobian.

#### Finite element model.

The finite element model represented the synthetic surface and concrete base as continuous solid domains, each corresponding to their respective material layers [24]. The domains were discretized into 3D solid elements and assembled to construct the full model. The impact load was applied using a rigid impactor, based on the artificial athlete Berlin Model, with contact defined over a circular area at the surface center. For simplification, the normal contact pressure was assumed to be uniformly distributed over the area, acknowledging that it may not fully capture the dynamic variation of real impact loading. The top surface of the model was assumed to be smooth and free of surface roughness or micro-cracks. The bottom of the concrete base was fully constrained to simulate fixed boundary conditions.

## Results and discussion

The force reduction (FR) defined in Eq (1) was used to estimate the shock absorption performance of the proposed semi-cast-in-situ surface. The FR might be influenced by various geometric parameters of the surface. To ensure the structural superiority of the surfaces, it was crucial to adhere to specific shock absorption threshold values. According to EN 14808 standards, a higher FR improved safety in protecting lower limbs during movement, provided that it did not exceed 50%. The subsequent analysis focused on the impact of variations in geometric parameters, aiming to offer insights for parameter optimization. The design parameters for synthetic sports surface were shown in Table 1.

**Table 1:** Essential parameters of the foundational model.

Work condition	Factor	Diameter of the holes (mm)	Shape of the holes	Distance between the holes (mm)	Arrangement of the holes	Thickness of the semi-cast-in-situ synthetic sports surface	
						Thickness of the casing layer (mm)	Thickness of the prefabricated layer (mm)
**1**	Shape of holes	30	Circular,Hexagonal and square	100	Square	12	6
**2**	Hole diameter	30,35,40,45,50	Circular	100	Square	12	6
**3**	Distance between holes	30	Circular	60, 70, 80, 90,100	Square	12	6
**4**	Arrangement of holes	30	Circular	100	Square, Equilateral triangular	12	6
**5**	Thickness of the synthetic layer	30	Circular	100	Square	10	8
						8	10
						6	12

### Effect of prefabricated hole shape on shock absorption of synthetic sports surfaces

**Fig 4:**
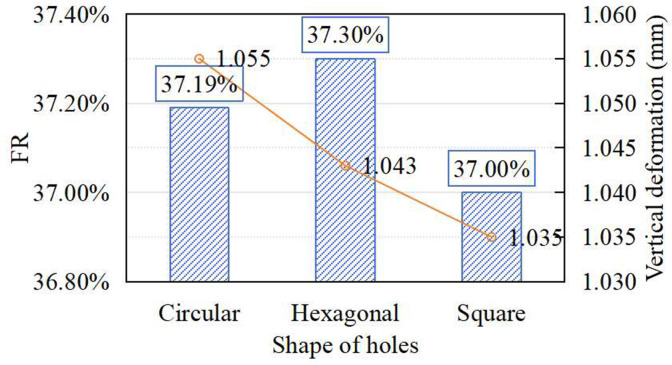
Effect of shape of prefabricated hole on FR and vertical deformation.

To evaluate the effect of prefabricated hole shape on surface performance, three geometries-circular, hexagonal, and square-were compared under controlled structural conditions: hole diameter (30 mm), spacing (100 mm), prefabricated layer thickness (6 mm), and cast-in-situ layer thickness (12 mm). As shown in Fig 4, FR values for all three hole shapes were nearly identical, averaging around 37%, with a maximum deviation of only 0.81%. The corresponding vertical deformation remained low, with a maximum of 1.055 mm. The results indicate that hole shape has minimal impact on the shock absorption performance within the tested configuration.

From a practical construction standpoint, circular holes are preferred due to their ease of fabrication, uniform stress distribution, and more consistent filling behavior. Their smooth geometry promotes complete material infusion, reducing voids and improving interlayer bonding. Compared to circular profiles, angular geometries such as square or hexagonal designs can impede material flow at corner regions, leading to incomplete filling, interface discontinuities, and elevated stress concentrations during loading. Therefore, while hole shape does not significantly affect mechanical response under ideal conditions, circular geometry offers construction and durability advantages, making it preferable for implementation.

### Effect of prefabricated layer thickness on shock absorption of synthetic sports surfaces

**Fig 5:**
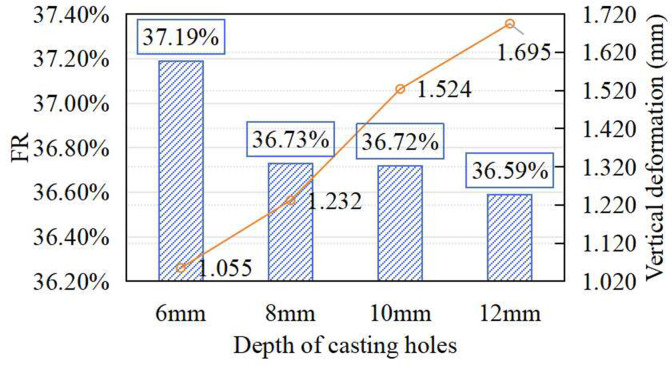
Effect of casting hole’s depth on FR and vertical deformation.

Fig 5 shows the effect of prefabricated layer thickness (i.e., casting hole depth) on the shock absorption and vertical deformation, with the hole shape fixed as circular (30 mm) and spacing set at 100 mm. As the layer thickness increased from 6 mm to 12 mm, the shock absorption capacity gradually declined. FR dropped by 1.23% as the thickness increased from 6 mm to 8 mm. A further increase to 10 mm produced negligible change, while FR at 12 mm decreased by 1.61% compared to the 6 mm configuration. Vertical deformation followed a similar trend. This decline in FR is attributed to increased hole depth reducing the relative volume of the cast-in-situ material in contact with the base, thereby altering energy transfer dynamics and making the structure stiffer under impact. The results suggest that a prefabricated layer thickness of 6 mm yields optimal shock absorption, consistent with the baseline design and within the EN 14808 standard range. This parameter choice balances energy dissipation and structural support effectively..

### Effect of prefabricated hole diameter on shock absorption of synthetic sports surfaces

**Fig 6:**
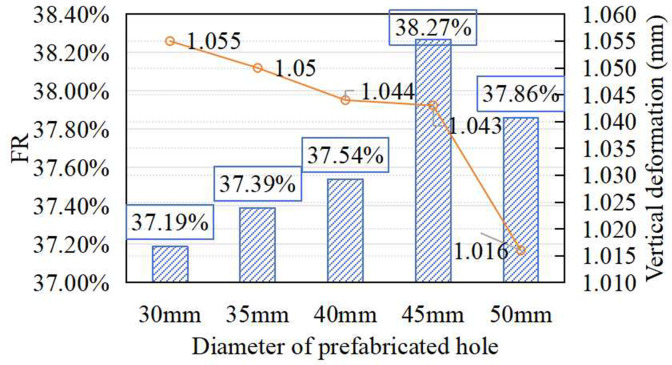
Effect of prefabricated hole diameter on FR and vertical deformation.

The impact of prefabricated hole diameter (30–50 mm) on shock absorption and surface deformation is shown in Fig 6. As the diameter increased from 30 mm to 45 mm, FR improved steadily, peaking at 38.27%, which is a 2.9% increase from the lowest value. That means that larger holes improve energy dissipation by reducing material stiffness beneath the impact zone. However, when the diameter reached 50 mm, FR declined noticeably, indicating that excessive hole size may weaken structural support and reduce impact attenuation efficiency. Vertical deformation decreased with increasing diameter, reaching a minimum at 50 mm, which suggests that large holes promote stiffness in deformation but at the expense of energy absorption. Based on the combined trend of FR and deformation, 45 mm was identified as the optimal hole diameter, balancing shock absorption and structural response.

**Fig 7:**
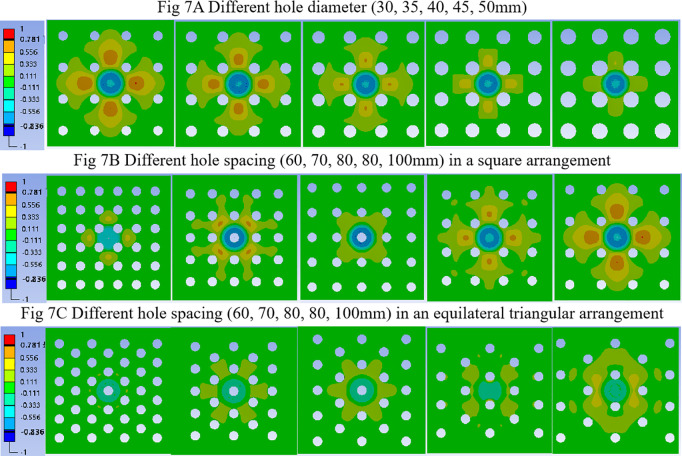
Deformation contours of synthetic sports surfaces with different hole diameter, hole spacing, and hole arrangement.

The deformation contours under instantaneous impact are given in Fig 7A. The deformation patterns were similar across all diameters, with uniform radial diffusion originating from a central “circular” area. The impact effect was effectively contained within the holes boundaries. As the diameter increased, the confining effect became more pronounced. The fundamental distribution pattern of impact loads remained unchanged across different diameter.

### Effect of holes spacing on shock absorption of synthetic sports surfaces

**Fig 8:**
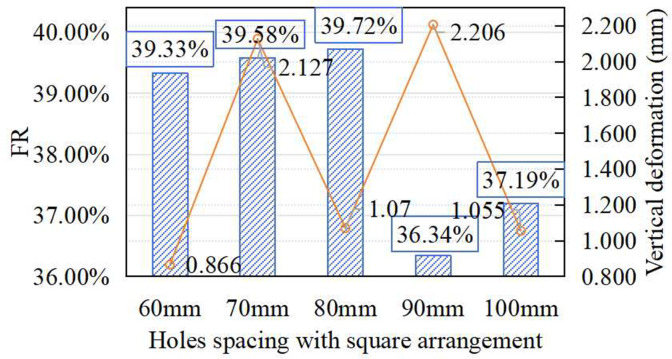
Effect of prefabricated hole spacing on FR and vertical deformation.

Fig 8 presents the effect of hole spacing (60–100 mm) on FR and vertical deformation, with the hole diameter fixed at 30 mm and a square arrangement maintained. FR decreased by 2.28% when the spacing was reduced from 100 mm to 90 mm, then sharply increased at 80 mm, reaching a maximum of 39.72%, which was 6.8% higher than the baseline value at 100 mm. Further reduction to 70 mm and 60 mm led to slight declines in FR. This V-shaped trend highlights the competing effects of hole density and structural stiffness on shock absorption performance. Vertical deformation followed a similar trend, with the minimum deformation also occurring at 80 mm. This alignment of minimal deformation and peak FR indicates that 80 mm achieves an optimal balance between energy dissipation and structural support.

Fig 7B illustrates how spacing holes affects the deformation field. At 100 mm spacing, the contour displayed a symmetric radial spread. When spacing was reduced to 80 mm, a casting hole aligns directly with the impact center, allowing efficient energy dispersion outward through four surrounding. At 60 mm, however, multiple holes concentrated within the central impact hole, restricting deformation transmission and reducing the effective load-bearing area. The results indicate that hole spacing not only alters the mechanical response but also reshapes the deformation transfer pathway, due to its direct influence on casting density and stress distribution.

### Effect of prefabricated hole arrangement on shock absorption of synthetic sports surfaces

**Fig 9:**
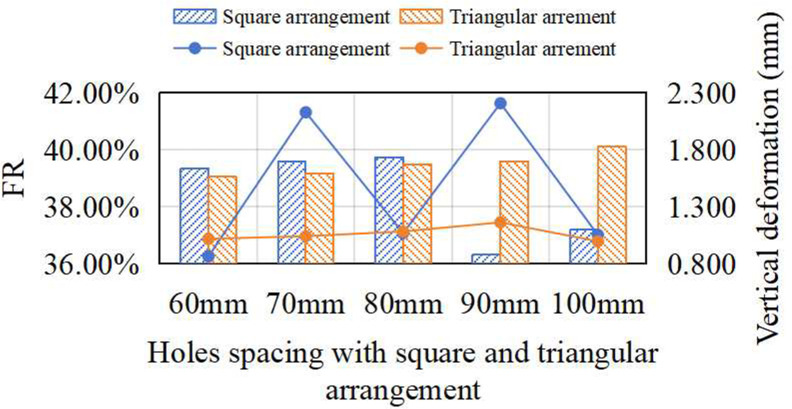
Effect of holes spacing with square and triangular arrangement on FR and vertical deformation.

Fig 9 compares the FR and vertical deformation of synthetic surfaces with equilateral triangular and square hole arrangements under varying spacing conditions. When the spacing was 100 mm or 90 mm, the triangular pattern achieved slightly higher FR than the square configuration. However, as the spacing decreased below 80 mm, the square pattern outperformed the triangular one. For both arrangements, peak FR was observed at a spacing of 80 mm and a hole diameter of 45 mm. Under these conditions, the square arrangement achieved a maximum FR of 42.34%, marginally higher than 41.17% for the triangular arrangement. Both values were significantly higher than those with 30 mm holes, indicating that arrangement effects are amplified at larger diameters. Vertical deformation showed minimal variation across different spacing under the triangular configuration. However, the underlying load transmission paths differed. At spacing of 100 mm and 90 mm, the presence of two adjacent holes at the force center restricted energy diffusion. In contrast, at 80 mm and below, a single hole was centered beneath the impactor, promoting more even outward load dispersion and resulting in a “six-pointed star” deformation contour. Compared with the square arrangement (Fig 7C), the triangular arrangement imposed more constraints on impact dispersion, particularly at smaller spacing, due to its denser center. That explains why the square arrangement exhibited slightly superior shock absorption at optimal spacing conditions.

### Effect of prefabricated layer multi-factors on shock absorption of synthetic sports surfaces

**Fig 10:**
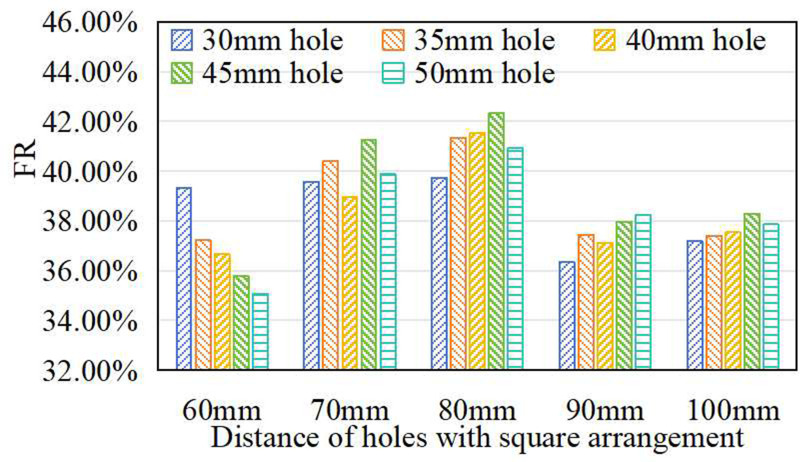
Effect of multi-factor of prefabricated layer on FR.

The combined effect of hole diameter and hole spacing on shock absorption was further examined to capture interaction effects beyond single-variable analysis. As shown in Fig 10, simulations were conducted under optimized baseline conditions: circular holes, a 6 mm prefabricated layer, a 12 mm cast-in-situ layer, and a square hole arrangement.

The results reveals a distinct “V-shaped” trend in FR as spacing decreased from 100 mm to 60 mm. In all cases, maximum FR was achieved at 80 mm spacing, regardless of hole diameter, reinforcing its role as a critical geometric parameter. However, when spacing became too small (e.g., 60 mm), FR declined, likely due to stress concentration and reduced structural continuity.

Although 80 mm was consistently optimal for spacing, the absolute FR values varied with hole diameter, indicating a nonlinear interaction between spacing and hole diameter. The peak FR of 42.23% was obtained at a holediameter of 45 mm and a spacing of 80 mm, representing a 13.85% improvement over the baseline condition (30 mm diameter, 100 mm spacing). These findings suggest that as spacing decreases, both the number and the total coverage area of casting holes increase in a nonlinear manner. This nonlinearity highlights the importance of simultaneously tuning hole density and size to balance energy dissipation, stiffness, and structural stability for optimal performance.

While the optimized configuration-45 mm hole diameter, 80 mm spacing, a 6 mm prefabricated layer, and a 12 mm cast-in-situ layer-demonstrates excellent shock absorption performance, it also balances engineering practicality. Further reducing spacing or increasing hole size would increase material usage and installation complexity, without significant improvement in performance. The semi-cast-in-situ design allows for modular and component-level repairability. Prefabricated panels can be selectively replaced without full-surface removal, thereby reducing long-term maintenance costs. From an environmental durability standpoint, the structure is more resilient to UV exposure, freeze–thaw cycles, and moisture accumulation. The presence of unbonded interfaces promotes vapor diffusion and drainage, helping prevent water-induced “heave” and extending service life under variable outdoor conditions.

## Model verification

**Fig 11:**
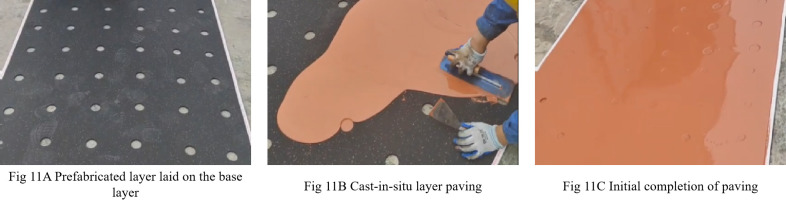
Laying process of semi-cast-in-situ synthetic sports field surface layer.

To validate the numerical simulation results under controlled conditions, a semi-cast-in-situ synthetic sports surface was fabricated using the previously identified optimal parameters: 45 mm circular holes, 80 mm spacing, a 6 mm prefabricated layer, a 12 mm cast-in-situ layer, and square hole arrangement. The test area was 800 mm × 2000 mm and was constructed on a standard concrete base (Fig 11).

### Mechanical properties of semi-cast-in-situ synthetic sports surfaces

The force reduction(FR) and the vertical deformation of the constructed surface were evaluated according to EN 14808. Test were conducted at multiple locations, and average values, were used. The measured FR was 41.38%, and the vertical deformation was 1.37 mm, both of which complied with EN 14808 requirements. The FR result deviated by less than 3% from the simulated value of 42.34%, confirming strong agreement between simulation and experiment. That high consistency effectively proved the validity of the numerical simulation results.

Mechanical properties were assessed via tensile testing of five cut samples using a WDW-1D microcomputer-controlled electronic universal machine, following the EN 14877 standard. All samples exceeded the minimum performance thresholds. The tensile strength ranged from 0.86 to 0.98 MPa, well above the 0.4 MPa requirement. And the elongation at break ranged from 78.2% to 89.6%, surpassing the 40% threshold. The results further confirm that the proposed surface design not only meets mechanical and safety standards but also demonstrates strong structural consistency with the numerical model.

## Conclusion and outlook

The study systematically evaluated the shock-absorption behaviour of semi-cast-in-situ synthetic sports surfaces through structural design, finite-element analysis, and experimental verification. An optimized configuration-45 mm circular holes, 80 mm spacing, a 6 mm prefabricated layer, and a 12 mm cast-in-situ layer arranged in a square pattern. This configuration achieved a force reduction of 42.23%, exceeding EN 14808 requirements, which effectively mitigated the risk of moisture-induced “heave”. Dry-condition field tests confirmed the simulation predictions: the measured force reduction was 41.38% and vertical deformation 1.37 mm, both within standard thresholds. These results support the structural feasibility and functional reliability of the proposed design in standard outdoor conditions.

Although moist-condition simulations were not included in the current study due to their experimental and modeling complexity, no signs of “heave” or surface cracking were observed in initial field trials. Future research will incorporate experimental and numerical validation under coupled temperature-humidity conditions, enabling a more comprehensive understanding of long-term performance. In addition, the present finite element model assumes a uniformly distributed normal pressure to represent the Berlin artificial-athlete loading. This simplification may not fully reproduce the dynamic evolution of the contact area. Future modeling will therefore incorporate pressure redistribution associated with varying contact geometry to improve simulation fidelity.

These present work contributes both theoretical insight and engineering guidance for the design and and optimization of environmentally adaptive synthetic track systems. The structural strategy proposed supports the development of surfaces that are safer, more durable, and more adaptable to environmental challenges, contributing to the sustainable advancement of the sports infrastructure industry.

## Supporting information

S1 File S1 Data. TensileTest_MooneyRivlin. S2 Fig. RawData_FR_Deformation_PrefabHoleShape. S3 Fig. RawData_HoleDepth_FR_Deformation. S4 Fig. RawData_HoleDiameter_FR_Deformation. S5 Fig. RawData_HoleSpacing_FR_Deformation. S6 Fig. RawData_HoleSpacing_SquareTriangular_FR_Deformation. S7 Fig. RawData_Multi-factor_FR_Deformation. S8 Validation Data. TensileTest_SyntheticSurface.
(ZIP)
